# A Specific Oligodeoxynucleotide Promotes the Differentiation of Osteoblasts via ERK and p38 MAPK Pathways

**DOI:** 10.3390/ijms13077902

**Published:** 2012-06-25

**Authors:** Xu Hou, Yuqin Shen, Chao Zhang, Liru Zhang, Yanyan Qin, Yongli Yu, Liying Wang, Xinhua Sun

**Affiliations:** 1 Department of Orthodontics, School of Stomatology, Jilin University, 1500 Qinghua Road, Changchun 130021, China; E-Mails: houxu@jlu.edu.cn (X.H.); zc11@mails.jlu.edu.cn (C.Z.); lrzhang10@mails.jlu.edu.cn (L.Z.); 2 Department of Periodontics, School of Stomatology, Jilin University, 1500 Qinghua Road, Changchun 130021, China; E-Mails: shenyq@jlu.edu.cn (Y.S.); yyqin09@mails.jlu.edu.cn (Y.Q.); 3 Department of Immunology, Medical College of Norman Bethune, Jilin University, Changchun 130021, China; E-Mail: ylyu@mail.jlu.edu.cn; 4 Department of Molecular Biology, Medical College of Norman Bethune, Jilin University, Changchun 130021, China; E-Mail: wlying@jlu.edu.cn

**Keywords:** oligodeoxynucleotide, osteoblast, differentiation, ERK1/2 MAPK, p38 MAPK

## Abstract

A specific oligodeoxynucleotide (ODN), ODN MT01, was found to have positive effects on the proliferation and activation of the osteoblast-like cell line MG 63. In this study, the detailed signaling pathways in which ODN MT01 promoted the differentiation of osteoblasts were systematically examined. ODN MT01 enhanced the expression of osteogenic marker genes, such as osteocalcin and type I collagen. Furthermore, ODN MT01 activated Runx2 phosphorylation via ERK1/2 mitogen-activated protein kinase (MAPK) and p38 MAPK. Consistently, ODN MT01 induced up-regulation of osteocalcin, alkaline phosphatase (ALP) and type I collagen, which was inhibited by pre-treatment with the ERK1/2 inhibitor U0126 and the p38 inhibitor SB203580. These results suggest that the ERK1/2 and p38 MAPK pathways, as well as Runx2 activation, are involved in ODN MT01-induced up-regulation of osteocalcin, type I collagen and the activity of ALP in MG 63 cells.

## 1. Introduction

Osteoblasts play an extremely important role in bone formation and remodeling processes. Osteoblasts are derived from mesenchymal stem cells, and can differentiate into mature and functional osteoblasts that produce extracellular matrix proteins and regulators of matrix mineralization [1]. Runx2 is an osteoblast-specific transcription factor that is essential for osteoblast differentiation and bone formation. Runx2^−/−^ mice show a complete lack of both intramembranous and endochondral ossification owing to maturation arrest of osteoblasts [2,3]. However, Runx2 can regulate the expression of osteoblast-specific genes including ALP, type I collagen and osteocalcin. To date, numerous reports have shown that signaling pathways and transcription factors could participate in osteoblastogenesis by regulating the production or activity of Runx2 [4]. Furthermore, Runx2 is necessary throughout life to promote the differentiation of new osteoblasts during bone remodeling [5].

Oligodeoxynucleotides (ODNs) containing unmethylated nucleotide motifs are immunostimulatory in vertebrates, and some ODNs containing CpG motifs are used for treating cancer, virus-associated diseases, and infections [6–9]. Recently, specific ODNs were found to have an effect on modulating osteoclast- and osteoblast-lineage cells [10]. In addition, CpG-ODN with a phosphorothioate backbone inhibits the BMP-induced phosphorylation of receptor-Smads in human mesenchymal stem cells and myeloma cell lines [11]. Chang *et al*. demonstrated that a novel CpG-ODN enhances the inhibitory effects on osteoclast differentiation by downregulation of TREM-2 [12]. In our previous studies, we have shown that ODN MT01, a synthetic 27-mer single stranded ODN with a design based on human mitochondrial DNA, promotes the proliferation and activation of osteoblasts [13], and stimulates the proliferation and differentiation of bone marrow mesenchymal stem cells (BMSCs) into osteoblasts [14]. However, the detailed signaling pathway in which ODN MT01 modulates the differentiation of osteoblasts has not been fully elucidated.

Mitogen-activated protein kinases (MAPKs) are a family of serine/threonine specific protein kinases, including p38, JNK and ERK1/2 (p44/p42). These molecules play important roles in cell differentiation, proliferation and death [15], and are activated by phosphorylation of tyrosine/threonine residues in signal transduction cascades. Previous studies have shown that the p38 MAPK and ERK1/2 pathways are involved in the proliferation and differentiation of osteoblasts [16–18]. Therefore, we hypothesized that ODN MT01 might regulate the differentiation of osteoblastic cells via the MAPKs pathway.

This study aimed to investigate the regulation of MAPKs in response to ODN MT01, and to elucidate the involvement of the MAPK signaling pathway in regulation of Runx2, osteocalcin, type I collagen and ALP expression in ODN MT01-treated MG 63 cells.

## 2. Results and Discussion

### 2.1. Internalization Analysis of ODNs

**Figure 1:**
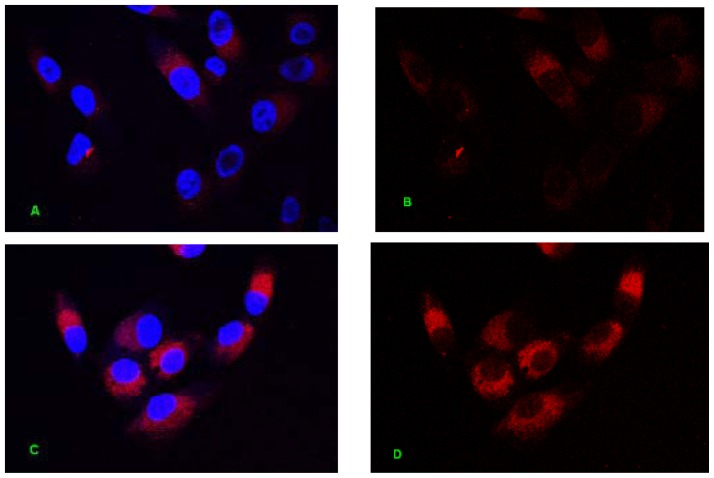

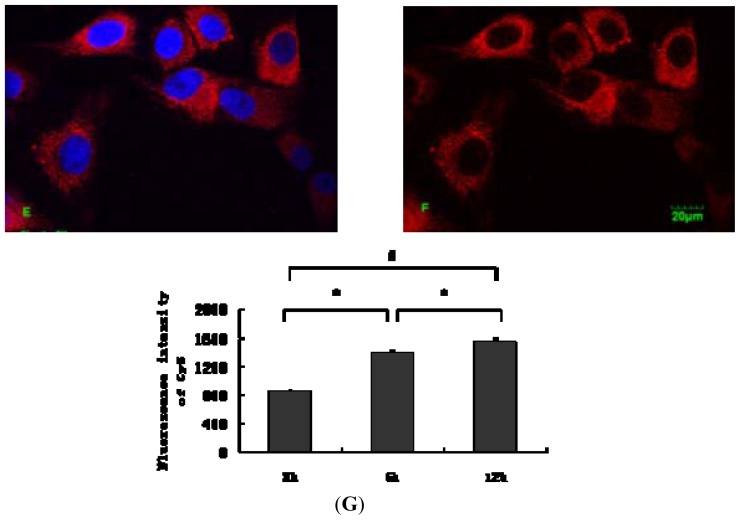
Confocal fluorescence images of MG 63 cells treated with Cy5-labeled MT01. (**A**,**C**,**E**) Merged images of Cy5-labeled MT01 and cells with Hoechst 33342-labeled nuclei; (**B**,**D**,**F**) Cy5-labeled MT01; (**A**,**B**) Cells were treated with MT01-Cy5 for 3 h; (**C**,**D**) Cells were treated with MT01-Cy5 for 6 h; (**E**,**F**) Cells were treated with MT01-Cy5 for 12 h. Red: Cy5-labeled MT01, Blue: nuclei. The fluorescence intensity of Cy5 was analyzed (**G**), * *p* < 0.05, # *p* < 0.01.

The design of ODN MT01, a 27-mer ODN, was based on the motif sequence 5′-ACCCCCTCTACCCCCTCT-3′ in human mitochondrial DNA. MT01 is a cytosine-rich ODN and contains five successive cytosines in each of its 9-mer motifs (5′-ACCCCCTCT-3′) [19]. To investigate whether the effects of an ODN required internalization into cells, Cy5-labeled ODN MT01 was synthesized to trace the sites of ODN actions. ODN internalization occurs spontaneously in culture without the need for uptake enhancers or transfection, and is temperature- and energy-dependent [20]. This result was consistent with our observations, as shown in Figure 1, ODN MT01 entered cells by endocytosis after incubation with MG 63 cells for 3 h. The fluorescence intensity was enhanced over time, and the intensity at 6 h was significantly higher than that at 3 h, and the intensity at 12 h was higher than that at 3 and 6 h. It was apparent that ODN MT01 stimulation of osteoblasts required internalization.

### 2.2. Effects of ODN MT01 on MAPK Signaling Proteins ERK1/2 and p38

**Figure 2:**
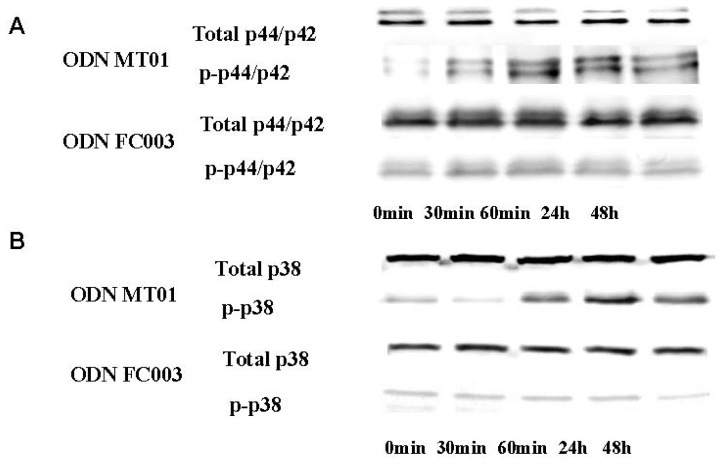
Western blot analysis of phosphorylated and total ERK1/2 (**A**), and total p38 (**B**). MG 63 cells were cultured in the presence or absence of 1 μg/mL ODN MT01 or ODN FC003. Cell lysates were obtained at 0, 30, 60 min, 24 h and 48 h after ODN treatment.

To investigate the effect of ODN MT01 on MAPKs, both total and phosphorylated levels of MAPKs were investigated by Western blotting after 0, 30, 60 min, 24 h and 48 h of treatment with 1 μg/mL ODN MT01. To better demonstrate that the effects of MT01 in MG 63 cells were in response to the specific sequence of MT01 we tested another ODN sequence, ODN FC003, a 24-mer ODN(5′-TCTCTCTCTCTCTCTCTCTCTCTC-3′). The design of ODN FC003 was also based on human mitochondrial DNA, and contained successive thymidine-cytosine nucleotides. In our previous studies [13,14], we found that ODN FC003 is inactive in the regulation of osteoblasts and bone marrow mesenchymal stem cells. Therefore, we chose this sequence as a negative control. As shown in Figure 2A, phosphorylation of ERK1/2 was observed after 30 min of treatment with ODN MT01, which increased by 13.1-fold after 60 min compared with that at 0 min. Furthermore, the active effect lasted up to 48 h (8.9-fold increase), while the total level of ERK1/2 protein remained unchanged. For p38, there was no obvious change in the phosphorylation level before 30 min. However, p38 phosphorylation increased after 60 min of treatment with MT01 (Figure 2B), and showed an increase of 4.7 fold at 24 h, which lasted up to 48 h. As shown in Figure 2 A and B, the FC003 sequence had no active effect on MG 63 cells via ERK1/2 or p38 MAPKs. Thus, these findings indicated that ODN MT01 induced the phosphorylation of ERK1/2 and p38 MAPK in MG 63 cells.

To investigate the mechanisms by which ODNs regulate the differentiation of osteoblasts, it is important to identify the functional role of signaling pathways activated by ODNs. At present, several signaling pathways have been shown to be involved in osteogenesis, such as MAPKs, BMP-2-Smad and NF-κB [21–23]. Among them, p38 and ERK1/2 MAPKs have been reported to be important for early osteoblast differentiation in various cell lines [24]. In this study, we found that MT01 was capable of activating ERK1/2 and p38 MAPK pathways indicating that MT01 induced the phosphorylation of MAPKs at the early stage of osteoblast differentiation. Previous reports have shown that ERK and p38 are phosphorylated in response to CpG-ODN, and p38 is phosphorylated to a lesser extent [25]. Thus, our findings were consistent with previous reports. ERK1/2 reacted more rapidly than p38 MAPK, and activation of both lasted up to 48 h after treatment with MT01. Once MAPK is activated by ODNs, transcription factors and other kinases may be phosphorylated to initiate events such as gene expression and post-translational protein modifications.

### 2.3. Effects of ERK and p38 Inhibitors on Up-Regulation of ALP Activity Induced by ODN MT01

To investigate the involvement of ERK and p38 MAPK in modulation of ALP activity induced by ODN MT01, specific chemical inhibitors of ERK and p38 (U0126 and SB203580, respectively) were used. Cells were pre-treated with or without 10 μM U0126 or SB203580 for 1 h, and then treated with or without 1 μg/mL ODN MT01 for 24, 48 and 72 h. As shown in Figure 2, compared with the control (phosphate-buffered saline (PBS) treatment), ALP activity was significantly up-regulated after ODN MT01 treatment at 48 and 72 h. Notably, U0126 and SB203580 significantly inhibited the up-regulation induced by ODN MT01.

**Figure 3:**
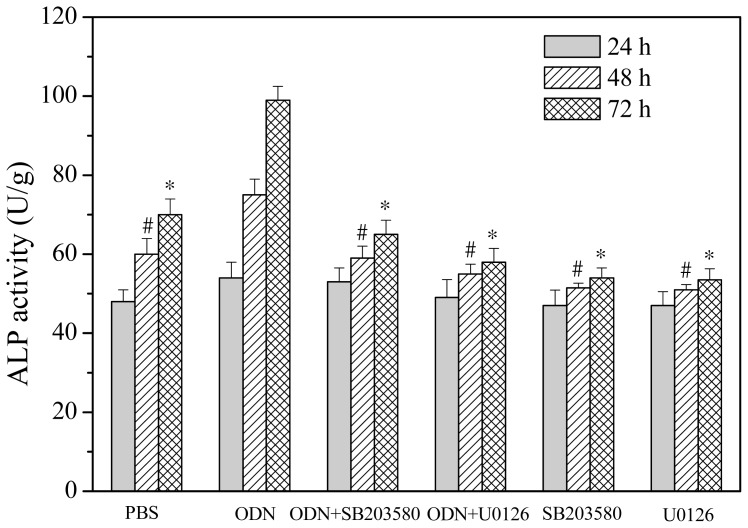
Effect of the ERK inhibitor U0126 (10 μM) and p38 inhibitor SB203580 (10 μM) on ALP activity in MG 63 cells after ODN MT01 (1 μg/mL) treatment for 24, 48 and 72 h. Data are expressed as the mean ± standard deviation (SD) (*n* = 6). # *p* < 0.05 *vs*. the corresponding value of ODN MT01 treatment at 48 h. * *p* < 0.05 *vs*. the corresponding value of ODN MT01 treatment at 72 h.

The activity of osteogenic differentiation marker ALP is an important index to indicate osteogenesis at an early stage. As shown in Figure 3, after 24 h of treatment with ODN MT01, the activity of ALP was increased, compared with that in the control, but the difference was not significant. The density of cells increased after 48 and 72 h, and ODN MT01 exhibited an obvious promoting effect on osteogenic differentiation. In addition, inhibitors significantly downregulated ALP activity induced by ODN MT01 after 48 and 72 h. These results indicated that the ERK and p38 MAPK pathways were probably involved in ODN MT01-induced promotion of ALP activity.

### 2.4. Effect of ERK and p38 Inhibitors on Runx2 Expression in Response to ODN MT01

**Figure 4:**
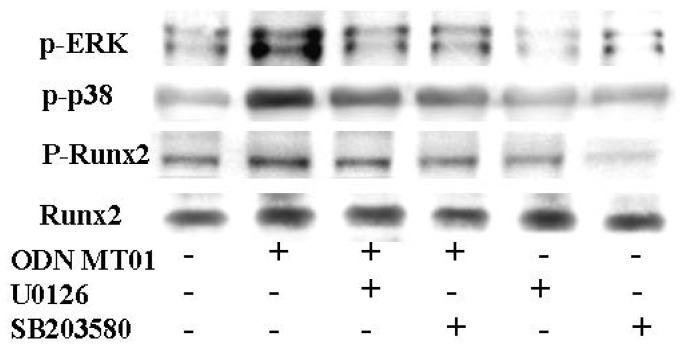
Effect of ERK and p38 inhibitors on Runx2 expression in response to ODN MT01. MG 63 cells were pre-treated with or without 10 μM U0126 or SB203580 for 1 h and then treated with or without 1 μg/mL ODN MT01 for 72 h. The protein expression of Runx2, p-Runx2, p-ERK and p-p38 was analyzed by Western blotting.

The above results confirmed that ODN MT01 induced the phosphorylation of ERK and p38 MAPK and further activated these MAPK signaling pathways. To investigate the role of Runx2, which is one of the most important factors for osteoblast differentiation, we used the ERK inhibitor U0126 and p38 inhibitor SB203580 to pre-treat cells for 1 h followed by treatment with 1 μg/mL ODN MT01 for 72 h. The total and phosphorylated protein levels of Runx2 were detected using Western blotting. As shown in Figure 4, there were obvious differences in the phosphorylation levels of ERK and p38 between the MT01-treatment and control groups; p-ERK and p-p38 were significantly inhibited in the presence of U0126 and SB203580, which were induced by MT01. For Runx2, there was no obvious change in protein levels in these groups, but MT01 induced notable phosphorylation of Runx2 (1.8-fold increase compared with the control). However, the up-regulated phosphorylation of Runx2 induced by MT01 was inhibited in the presence of U0126 and SB203580.

Runx2 is an essential transcription factor for osteoblast differentiation and bone formation, and inactivation of one Runx2 allele will cause cleidocranial dysplasia syndrome, a disease characterized by delayed osteoblast differentiation for bone formation through intramembranous ossification in mice and humans [3,26]. Previous reports have shown that extracellular matrix production can induce osteoblast differentiation by increasing Runx2 transcriptional activity, but not the mRNA or protein levels [27–29], which concurs with our results. Runx2 activity is controlled by various extracellular signaling pathways, and post-translational modifications (phosphorylation, acetylation and ubiquitination) can affect its stability and activity [30,31]. Among the post-translational modifications, the role of Runx2 phosphorylation has been well characterized. Ge *et al*. found that Runx2 is regulated by ERK1/2 and p38 MAPK-mediated phosphorylation [32], and ERK is more important for Runx2 phosphorylation than p38 [33]. Interestingly, in the present study, we found the same results in which inhibition of ERK showed a stronger effect on the expression of p-Runx2 (1-fold increase compared with the control) than that of p38 inhibition (1.2-fold increase compared with the control). These results suggested that Runx2 is phosphorylated on a complex set of sites that partially overlap between ERK and p38. Another study found that phosphorylation of p38 MAPK induces activation of Runx2 via TAK1 and MEK3 signaling pathways during early osteoblastic differentiation [21]. Here, we showed that MT01 greatly increased Runx2 phosphorylation, which was potently repressed by inhibitors of ERK and p38. Taken together, our results were consistent with other findings and demonstrated that MT01-induced phosphorylation of Runx2 was mediated by activation of both ERK1/2 and p38 MAPK signaling pathways, and ERK MAPK may be more important.

### 2.5. Effects of ERK and p38 Inhibitors on the Expression of Osteocalcin and Type I Collagen

**Figure 5:**
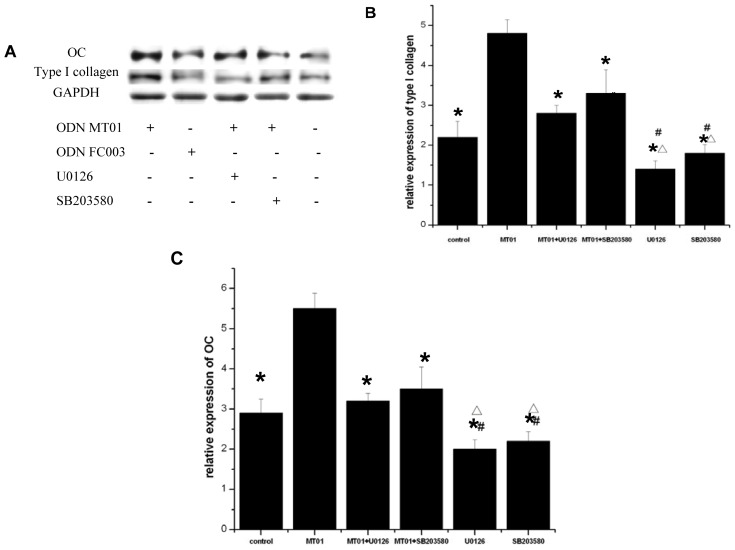
MG 63 cells were pre-treated with or without 10 μM U0126 and SB203580 for 1 h and then treated with or without 1 μg/mL ODN MT01 for 15 d. Another group was treated with 1 μg/mL ODN FC003. Medium was changed every 3 days, and ODNs and inhibitors were added as the same time. (**A**) Western blot analysis of osteocalcin (OC) and type I collagen protein expression; (**B**) Real-time PCR analysis of type I collagen mRNA expression; (**C**) Real-time PCR analysis of OC mRNA expression. The gene expression of type I collagen and OC was measured by real-time PCR and results were normalized against the average housekeeping gene expression in each sample. One-way ANOVA and the Bonferroni *post-hoc* test were used to compare differences between the ODN MT01-treated group and other groups. * *p* < 0.05 *vs*. the corresponding value of ODN; Δ *p* < 0.05 *vs*. the corresponding value of ODN+U0126; # *p* < 0.05 *vs*. the corresponding value of ODN+SB203580. Data are expressed as the mean ± SD (*n* = 6).

Runx2 has been shown to regulate the expression of osteoblast-specific genes, such as ALP, osteocalcin and type I collagen [34]. To further confirm our results, we analyzed the expression of subsets of osteogenic marker genes including osteocalcin and type I collagen. We used ERK and p38 inhibitors to pre-treat cells for 1 h followed by treatment with 1 μg/mL ODN MT01 or ODN FC003 for 15 days. Then, the mRNA and protein expression of osteocalcin and type I collagen were analyzed by real-time PCR and Western blot, respectively. As shown in Figure 5A, compared with the control g, the protein expression of osteocalcin was increased by ODN MT01 treatment. Compared with ODN MT01 treatment, the protein level of osteocalcin was significantly decreased when cells were pretreated with the ERK and p38 inhibitors. The expression level of type I collagen exhibited a similar tendency with osteocalcin. As shown in Figure 5B,C, the mRNA levels were concurrent with the protein expression.

Osteocalcin protein expression marks the late phase of osteogenic differentiation, and is only produced by mineralizing cells types. In addition, osteocalcin is considered as a specific marker of osteoblast differentiation and maturity. Type I collagen is the major product of osteoblasts, and accounts for 90% of the matrix protein content [35]. Type I collagen genes are expressed in osteoblastic cells at all stages during development and throughout life. Osteocalcin and type I collagen protein expression induced by ODN MT01 was increased at late stages of osteoblast differentiation, suggesting that ODN MT01 controll of the expression of osteocalcin and type I collagen in these cells could also control osteoblast differentiation and function. Furthermore, ERK and p38 MAPK inhibitors obviously decreased protein expression induced by MT01. Therefore, ODN MT01 induced the expression of osteocalcin and type I collagen via ERK and p38 MAPK signaling pathways.

## 3. Experimental Section

### 3.1. Materials

ODNs MT01 (5′-ACCCCCTCTACCCCCTCTACCCCCTCT-3′) and FC003 (5′-TCTCTCTCTCTC TCTCTCTCTCTC-3′) were synthesized by TaKaRa (Dalian, China), and were dissolved in axenic PBS. The human osteoblastic cell line MG 63 was obtained from the American Type Culture Collection (CRL-1427). An Alkaline phosphatase kit and micro-BCA assay kit were obtained from Jiancheng Biological Reagent Co. (Nanjing, China). A real-time PCR kit was purchased from TaKaRa (Tokyo, Japan). Monoclonal antibodies against p38, p-p38, p44/p42 and p-p44/42, were purchased from Santa Cruz Biotech. and those against Runx2, osteocalcin and type I collagen were purchased from Abcam (UK). The anti-phospho-Runx2 antibody was purchased from Abcam Co (UK). The phosphorylation site was Ser533.

### 3.2. Cell Culture

MG 63 cells were cultured in Dulbecco’s modified Eagle’s medium (Sigma, St. Louis, MO, USA) containing 10% heat-inactivated fetal calf serum, 100 U/mL penicillin and 100 mg/mL streptomycin at 37 °C and 5% CO_2_. Cells were seeded at an initial density of 2 × 10^5^ cells/mL. To investigate the effect of ODN MT01 on MAPKs, cells were treated with 1 μg/mL ODN MT01 for 0, 30, 60 min, 24 and 48 h. To investigate activation of Runx2, cells were pre-treated with or without the ERK inhibitor U0126 (10 μM) and p38 inhibitor SB203580 (10 μM) for 1 h, and then treated with or without ODN MT01 for 72 h. To investigate the effects of ERK and p38 inhibitors on the mRNA and protein levels of osteocalcin and type I collagen, cells were pre-treated with or without the ERK inhibitor U0126 (10 μM) and p38 inhibitor SB203580 (10 μM) for 1 h, and then treated with or without ODN MT01 for 15 days. Medium was changed every 3 days, and ODNs and inhibitors were added at the same time.

### 3.3. Fluorescent Labeling of MT01

Cy5 was conjugated to the 5′-end of MT01, and then dissolved with PBS. Cells were cultured on 24 × 24 mm coverslips at a density of 1 × 10^4^ cells/well overnight. Then, cells were treated with 1 μg/mL Cy5-labeled MT01 for 3, 6 and 12 h. Cells were washed three times with PBS according to the time points. While protected from light, Hoechst 33342 was added at 1 μg/mL for 5 min to stain nuclei. A laser scanning confocal microscope (Olympus IX81, Japan) was used to observe cell climb-chips. Stained cells were imaged with Olympus Fluoview FV1000 Viewer (Version1.7.c; Olympus Corporation: Tokyo, Japan, 2007).

### 3.4. Western Blot Analysis

Western blotting was used to evaluate total and phosphorylated protein levels of p38 and p44/42. MG 63 cells were cultured in 10-cm dishes and treated with 1 μg/mL ODN MT01 for the indicated times. To evaluate the protein levels of Runx2 and p-Runx2, cells were pre-treated with or without 10 μM U0126 or SB203580 for 1 h and then treated with or without ODN MT01 for 72 h. To investigate the levels of osteocalcin and type I collagen, cells were pre-treated with or without 10 μM U0126 or SB203580 for 1 h, and then treated with or without ODN MT01 for 15 days. Then cells were collected, washed twice with cold Tris-buffered saline and resuspended in lysis buffer (50 mM Tris, pH 7.6, 0.01% EDTA, 1% Triton X-100, 1 mM PMSF, and 1 μg/mL leupeptin). The protein concentration was measured using a BCA Protein Assay Reagent kit. Protein samples (40 μg) were separated by 10% SDS-PAGE and then transferred to polyvinylidene difluoride (PVDF) membranes. The membranes were blocked with 5% skim milk powder in Tris-buffered saline with 0.1% Tween (TBST) for 1 h at room temperature, followed by incubation with primary antibodies at 4 °C overnight. Then membranes were incubated with secondary antibodies at 20 °C for 2 h, and developed using an ECL chemiluminescent system. Loading differences were normalized using a GAPDH antibody.

### 3.5. ALP Activity Assay

Cells were pre-treated with or without 10 μM U0126 and SB203580 for 1 h and then treated with or without 1 μg/mL ODN MT01 for 24, 48 and 72 h. Then, the cells were collected and lysed to measure ALP activity. The assay was conducted using an Alkaline Phosphatase Kit, according to the manufacturer’s instructions. The protein concentration of cell lysates was measured using a micro-BCA assay kit, and ALP activity was normalized to the total protein concentration. Values were the averages of triplicate measurements.

### 3.6. Real-Time PCR

To quantify mRNA expression levels, real-time PCR was performed with cDNA samples. Primers were designed using qPrimerDepot(Nucleic Acids Res. 2007), a primer database for quantitative real-time PCR. Real-time PCR was performed using an ABI Steponeplus (ABI PRISM, Carlsbad, NM, USA), which allowed real-time monitoring of increases in PCR product concentrations after each cycle based on the fluorescence of the double-stranded DNA specific dye SYBR green. The number of cycles required to produce a detectable product above background was measured for each sample. These cycle numbers were then used to calculate fold differences in the initial mRNA level for each sample using the following method. First, the cycle number difference for GAPDH, a housekeeping gene, was determined in the control sample and appropriate ODN MT01-treated sample. Values were the averages of triplicate measurements. PCR primers used were as follows: GAPDH forward, 5′-GAAGGTGAAGGTCGGAGTC-3′ and reverse, 5′-GAAGATGGTGATGGGATTTC-3′; Type I collagen forward, 5′-AGGGCCAAGACGAAGACATC-3′ and reverse, 5′-AGATCACGTCATCGCACAACA-3′; OC forward, 5′-TGAGAGCCCTCACACTCCTC-3′ and reverse, 5′-GCCGTAGAAGCGCCGATAGGC-3′.

### 3.7. Statistical Analysis

All experiments were performed with triplicate independent samples and repeated at least twice. Results were expressed as the mean ± SD. ANOVA and the Bonferroni *post-hoc* test were used to compare the differences between the ODN MT01-treatment group and other groups. A value of *p* < 0.05 was considered statistically significant. Statistical analysis was performed with SAS software (version 8.0, SAS: Cary, NC, USA).

## 4. Conclusions

In this study, a specific ODN, ODN MT01, was endocytosed by osteoblasts and found to up-regulate the expression level of osteocalcin, type I collagen and ALP in MG 63 cells via the ERK1/2 and p38 MAPK pathways. This study provides further insight into the use of ODN MT01 for *in vitro* experimentation, and supports the potential use of ODN MT01 to regulate the rebuilding of bone.
